# Agreement between questionnaire and medical records on some health and socioeconomic problems among poisoning cases

**DOI:** 10.1186/1756-0500-2-183

**Published:** 2009-09-14

**Authors:** Ahmed I Fathelrahman

**Affiliations:** 1 National Poison Center, Universiti Sains Malaysia, Minden, 11800 USM Pulau Pinang, Malaysia

## Abstract

**Background:**

The main objective of the present study was to evaluate the agreement between questionnaire and medical records on some health and socioeconomic problems among poisoning cases.

**Methods:**

Cross-sectional sample of 100 poisoning cases consecutively admitted to the Hospital Pulau Pinang, Malaysia during the period from September 2003 to February 2004 were studied. Data on health and socioeconomic problems were collected both by self-administered questionnaire and from medical records. Agreement between the two sets of data was assessed by calculating the concordance rate, Kappa (k) and PABAK. McNemar statistic was used to test differences between categories.

**Results:**

Data collected by questionnaire and medical records showed excellent agreement on the "marital status"; good agreements on "chronic illness", "psychiatric illness", and "previous history of poisoning"; and fair agreements on "at least one health problem", and "boy-girl friends problem". PABAK values suggest better agreements' measures.

**Conclusion:**

There were excellent to good agreements between questionnaire and medical records on the marital status and most of the health problems and fair to poor agreements on the majority of socioeconomic problems. The implications of those findings were discussed.

## Background

The agreement between medical records and data collected by questionnaire has been evaluated extensively on various ailments and health conditions [1-8]. Those studies revealed wide variations in findings ranging from perfect agreements to almost no agreements [9,10]. Medical records have the limitations of being incomplete, missed, conflicting or irrelevant [11]. A record in one health setting does not cover all the information required by the investigator, and patients normally seek treatment from several clinicians. Moreover, medical records are not written for the purposes of the specific study. On the other hand, data collected by questionnaire is also fraught with other limitations such as denial, difficulties in retrieval, telescoping, hindsight, and other recalling problems [12].

There are situations where researchers face the need to use data composed of information collected in part retrospectively (from medical records) and in part prospectively (by questionnaire) may be as an attempt to increase sample size or to complete missing information in one source [13-15]. This may raise questions about the validity of findings based on concerns about the agreement between the data collected by different methods.

Acute poisoning is a significant health problem that has been studied widely. However, to our knowledge there has been little or no work evaluating methods of data collection in poisoning documents.

Previous studies on poisoning in Malaysia have used different sources of data collection including hospital records [16,17], poison center records [18], national database [19] and questionnaire [20]. In a work reported elsewhere, we used data collected by questionnaire to complete missing information in medical records [21]. This led us to think about the agreement between the two instruments on data related to poisoning and then to conduct this study.

The objective of the present study was to evaluate the agreement between questionnaire and medical records on some health and socioeconomic problems among poisoning cases admitted to Penang general Hospital during the period from September 2003 to February 2004. The researcher was interested also to test the assumption that a specific questionnaire would be expected always to provide greater positive response rate for questions being asked than physician interviews documented in medical records.

## Methods

### Study design and patients' characteristics

The present work is a secondary analysis of data on poisoning reported previously in a case-control study [20]. 100 patients admitted consecutively with drug overdose or chemical poisoning to the Hospital Pulau Pinang during the period from September 2003 to February 2004 were studied. The sample size was calculated for the purpose of the primary study to achieve a significance level of 0.05 and a power of 80%, based on an odds ratio of 2 [20]. Patients who were treated at the emergency room and not admitted were not included since they are normally discharged within few hours of presentation to the hospital with a greater difficulty to be catch and surveyed. Furthermore, those patients do not have good and complete data in the medical records. Patients who were admitted for food poisoning, adverse drug reactions, and drug- related interactions, poisonings with plants and animal venoms and chronic exposures such as digoxin and warfarin toxicities were also excluded. Patients, who died, absconded, discharged before being contacted by the researcher or refused to participate in the study, were considered non-respondents. During study period 32 poisoning cases were excluded and 20 cases were non-respondent (response rate = 83.33%). Of cases responded, 70% were female and 61% were aged 20 to 39 years old (Table 1). More details about the characteristics of those poisoning cases were described in the previous work [20].

**Table 1:** Demographic characteristics of the 100 poisoning patients

**Variable**	**Categories**	**Frequency (%)**
Age groups	≤ 19	17 (17)
	20-29	39 (39)
	30-39	22 (22)
	40-49	14 (14)
	50-59	3 (3)
	>60	3 (3)
Ethnic group	Chinese	34 (34)
	Indians	34 (34)
	Malay	28 (28)
	Other	4 (4)
Gender	Female	70 (70)
	Male	30 (30)
Highest education	Primary	16 (16)
	Secondary	66 (66)
	Tertiary	11 (11)
	None	7 (7)

### Ethical considerations and questionnaire validation

The study was approved by the Hospital Research Committee, and patients have been consented using written consent forms. The questionnaire and the consent form were written in English and Malay and were validated by translation and back translation. Both of the English and the Malay questions were printed in the same questionnaire rather than as two separated copies. A pilot study was done using the first 30 poisoning cases. Our patients faced no problems in understanding, self-administering and answering the questionnaire, and it was accepted without any modifications. Patients' medical records were written in English and items included in the data collection form were also written in English.

### Data collection

The data on socioeconomic and health problems were collected during patients' hospitalization using self-administered questionnaire. Patients were asked to report any socioeconomic and/or health problem since two months before their current admission as well as previous events of poisoning (including any past history of poisoning regardless whether the patient was presented to the hospital, self-treated at home or treated at a health facility elsewhere). For the purpose of the primary study, the questionnaire was designed to collect information on socioeconomic status (such as employment and total family income), social environment (such as cohabitation and number of persons in the same household), health environment (such as illness among other family members), as well as toxic agents' availability and accessibility (such as storage practice of drugs and chemicals). However, in this secondary analysis, we included only the marital status and the socioeconomic and health problems since they are relatively well documented by the medical records. Socioeconomic problems included problems related to academic, family, marital, parental, work, boy-girl friends' relationships, financial and others. Health problems included chronic illness, recent physical illness, and psychiatric illness. Each one of the previous items was asked about separately using close- ended questions (structured) that were provided by two possible answers (yes or no). No specific definitions were given to the patients for the terms like "academic problems" or "family problems". This is because a preliminary study conducted at the same hospital using patients' records identified the common use of such terminologies. Moreover, the pilot study carried in the beginning of this work revealed familiarity and well understanding of patients to what was meant by those problems. The choice of the "family problems" was offered to cover problems others than the marital and the parental since the families in the Malaysian community are big extended families that include more than the parents, spouse and children. Furthermore, some patients who may be reluctant to elaborate clearly about the nature of a family problem may prefer to describe those problems as family-related rather than marital or parental. After patient discharge, the same information mentioned above was extracted by the researcher from patients' medical records using specially designed data collection forms.

### Certain considerations

In the current work, when the specific health problem or socioeconomic problem was not reported in medical records, we assumed that it didn't occur (i.e. the answer was considered No). This is because the medical records data seldom report negative responses for the items studied (e.g. reporting that there was no history of previous poisoning or no history of a psychiatric problem) except for cases suspected to be suicidal attempts. Nevertheless, our questionnaire required that a patient provides a clear response (positive or negative).

The marital status was a three-level categorical variable, married, single, and divorced or widowed (i.e. concordance rates and kappa values were computed from 3 × 3 table); whereas, all other variables studied were dichotomous (i.e. concordance rates and kappa values were computed from 2 × 2 tables).

### Statistical analyses

The agreement between the two sets of data was assessed by calculating the concordance rate, Kappa (k) and PABAK values. Concordance rate (crude agreement) is equal to the sum of instances reported and instances not reported by both data divided by total number of instances studied [22]. Kappa is an agreement's measure beyond that agreement expected to occur by chance. Kappa values were computed using the Statistical Package for the Social Sciences (SPSS) software and were interpreted according to the suggestions provided by Byrt [23]; which are 0.93-1.00 for excellent, 0.81-0.92 (very good), 0.61-0.80 (good), 0.41-0.60 (fair), 0.21-0.40 (slight), 0.01-0.20 (poor), and 0.00 or less for no agreement. PABAK states for the Prevalence and Bias Adjusted Kappa, and its values were calculated by the equation PABAK = 2*P*_*o*_-1 [24], where *P*_*o *_is the observed agreements and is equal to (a+ d)/n.

Comparative analyses were done using McNemar test when applicable. P value of < 0.05 was considered statistically significant.

## Results

Crude agreements between medical records and questionnaire data ranged from 62% for "at least one socioeconomic problem" to 96% for "marital status", with the majority of variables showing agreements higher than 70% (Table 2).

**Table 2:** Agreements between medical records and questionnaire on socioeconomic and health problems among poisoning patients

**Socioeconomic and health variables**		**Q** **Freq (%)**	**M** **Freq (%)**	**P value^a^**	**Concordance** **rate^b^**	**Kappa (*95% CI*)**	**PABAK (*95% CI*)**
Marital status	Married	39 (39)	30 (30)	>0.999	96%	0.93 (0.833 - 1.025)	0.92 (0.843 - 0.997)
	Single	55 (55)	20 (20)				
	Divorced or widowed	6 (6)	3 (3)				
Health problems	Chronic illness	18 (18)	13 (13)	0.180	91%	0.66 (0.454 - 0.862)	0.82 (0.708 - 0.932)
	Recent illness	18 (18)	5 (5)	0.011	77%	-0.08 (-0.146 - -0.024)	0.54 (0.375 - 0.705)
	Psychiatric illness	9 (9)	14 (14)	0.125	93%	0.66 (0.427 - 0.889)	0.86 (0.760 - 0.960)
	At least one problem	37 (37)	31 (31)	0.307	76%	0.47 (0.287 - 0.647)	0.52 (0.353 - 0.687)
Socioeconomic problems	Academic	7 (7)	4 (4)	0.453	93%	0.33 (-0.042 - 0.702)	0.86 (0.760 - 0.960)
	Family	27 (27)	16 (16)	0.052	73%	0.21 (0.006 - 0.422)	0.46 (0.286 - 0.634)
	Marital	9 (9)	13 (13)	0.424	86%	0.29 (0.014 - 0.562)	0.72 (0.584 - 0.856)
	Parental	7 (7)	9 (9)	0.754	90%	0.32 (0.004 - 0.639)	0.80 (0.682 - 0.918)
	At work	7 (7)	2 (2)	0.180	91%	-0.03 (-0.067 - 0.003)	0.82 (0.708 - 0.932)
	Boy-girl friends	13 (13)	10 (10)	0.549	89%	0.46 (0.192 - 0.729)	0.78 (0.657 - 0.903)
	Financial	5 (5)	5 (5)	>0.999	92%	0.16 (-0.185 - 0.501)	0.84 (0.734 - 0.946)
	Other problems	5 (5)	4 (4)	>0.999	91%	-0.05 (-0.078 - -0.016)	0.82 (0.708 - 0.932)
	At least one problem	61 (61)	53 (53)	0.256	62%	0.23 (0.042 - 0.418)	0.24 (0.050 - 0.430)
Previous poisoning	(yes)	11 (11)	6 (6)	0.063	95%	0.68 (0.422 - 0.940)	0.90 (0.814 - 0.985)

Q: questionnaire; M: medical records; Freq: frequency; ^a ^McNemar test, ^b ^Concordance rate (crude agreement) = number of instances reported by both data + number of instances not reported by both data/total number studied.

By comparing different categories of marital status, there was clear discrepancy between data collected by questionnaire and that collected from medical records. A significant proportion (47%) of medical records did not report the marital status (i.e. missing information); the majority (74.5%) of them (according to the data collected by questionnaire) was singles. However, assessing the agreement between the two sets of data on the completed records revealed excellent agreement (Kappa = 0.93). In fact marital status showed the highest agreement among studied items.

The data about each of "chronic illness" (kappa = 0.66), "psychiatric illness" (kappa = 0.66), and "previous history of poisoning" (kappa = 0.68) showed good agreements between questionnaire and records, whereas, "at least one health problem" (kappa = 0.47), and "boy-girl friends problem" (kappa = 0.46) showed fair agreements. Those kappa values were statistically significant (p < 0.05). PABAK values indicated fair to excellent agreements on most items except "at least one socioeconomic problem".

The medical records were found to be significantly less likely than questionnaire to report "recent illness" (p values < 0.05). There were no further significant differences between the medical records data and the questionnaire in reporting other health and socioeconomic problems.

## Discussion

Medical records are completed at the emergency room by physicians relying on the report of patient under the stress of poisoning exposure, suffering from pain, fearing from treatment [25], and that may well be confused especially if he is sedated by a drug or alcohol. Moreover, physicians are not expected to write about an illness unless it is directly linked to the poisoning episode and clearly identified or supported by documented evidence. On the other hand, patient completes questionnaire within 24 hours later, not under the influence of the poison, may over-report problems in order to seek attention or shift the attention from recent events.

Those factors may explain way questionnaire provides greater positive response rate for most of questions being asked than physician interviews documented in medical records [26]. Although statistically not significant, psychiatric events have been reported more frequently by medical records than questionnaire. Reporting psychiatric events by medical records were not based on self-report; rather they were based on actual medical history and true admissions, follow-up, and or received treatments. The patients might be unaware of their psychiatric morbidities, or were not considering them as psychiatric illnesses. Moreover, poisoning patients were unlikely to over report psychiatric problems since psychiatric morbidities may be considered by the community as a stigma. Janson [12] concluded that patients tend to over report presumably socially desirable aspects and under report socially undesirable aspects.

The questionnaire and medical records have shown excellent agreement on the "marital status". This is despite a wide discrepancy between them in frequencies (the status of almost half of the cases was not reported in the medical records). The majority of those missed classifications were singles (74.5%). This highlights the issue of missing and incomplete data in the medical records. Nagurney et al [11] concluded that the information obtained from medical records is measurably less accurate than information obtained by questionnaire. For certain items, more than half of the information is not available. Nagurney and associates discussed the importance to use templates with checklists by the clinicians while writing medical charts to ensure that negative data are checked as such, rather than appearing as missing [11].

In our present work, the agreements between medical records and questionnaire were found to be good on "chronic illnesses" and "previous history of poisoning", but poor on "recent illness". Similar literature revealed good to substantial agreements between self-reported information (questionnaire) and medical records on common chronic conditions [3,10] and poor agreements on uncommon chronic conditions, acute illnesses and those with less explicit diagnostic criteria [3,9,26].

In the present work, each of the data reported by medical records and by questionnaire is a self-report; however timings of the two self-reports were different. In the first case, patients were reporting to physician at emergency room or directly after admission, whereas in the second case (questionnaire), patients were reporting after completing treatment or at least after their medical condition were stabilized (within 24 hours of admission). In the previous literature, patients self-report seemed to be affected by several factors [8,26,27] such as patient's characteristics, situation of patient at time of reporting, type of information required to be recalled, vividness of the reported exposures and events (i.e. major life events versus minor ones), and time of interview. Neugut and Neugut [25] found that relatively low proportion of patients could accurately tell the physician in the emergency room about the reason for their prior admission. The authors related this partially to the stresses at the emergency room.

Our findings have shown poor agreements on the whole socioeconomic problems except "boy-girl friend relationships". Generally, socioeconomic problems have been reported by questionnaire more frequently (61.4%) than being reported by medical records (53.5%). This is also may be due to over reporting of those problems by the poisoned patients during answering questionnaire, for attention seeking.

The lower kappa values in a lot of items were not associated with lower concordance rates a phenomenon widely discussed in the previous literature that is attributed to the prevalence effect [24,28,29]. In a case of rare finding, very low kappa does not merely reflect lower agreement. Moreover, the values of PABAK, a corrected measure of kappa, reflect excellent to fair agreements in most items with low kappa.

One of the possible reasons for the very low kappa values, particularly in regard to the socioeconomic problems, is our assumption that characteristics not reported in medical records are absent (i.e. negative). Parts of those assumed to be negative responses were actually positive responses ignored by the treating physician. One of the challenges that faced the researcher was whether to consider the data in such situation as missing or as negative responses. If the first approach was used, greater proportions of the studied data would be missed because the medical records seldom report negative responses for the items studied without strong justification. The researcher selected the first choice with regard to the marital status only since there was no chance to consider the lack of information about this parameter as a negative response (i.e. if it was not "single" it would certainly be "married" or "divorced or widowed"). The so many missing data about marital status in medical records has reduced the total number of the valid cases to 53 out of the 100. Nagurney et al coded the items which were not positively stated in the medical records as negative, implied as negative, uncertain, or missing [11].

It is difficult to assume superiority of either source of information (questionnaire & medical records). This is because each one is subjected to different factors of strengths and weaknesses [11]. While discussing their findings, Zhu and associates suggested the use of the two sets of data interchangeably whenever there is a high concordance as so as missing values from one source can be replaced with those from the other [26]. Yoon et al reported that some data elements used for acute stroke care registry are better collected prospectively and others retrospectively [14]. Moreover, they concluded that combined retrospective and prospective data-collection methods may be best in terms of both completeness and accuracy. In the same context, we suggest that in common and chronic health conditions and major life events (such as previous poisoning), where both of medical records and questionnaire are expected to provide highly concordant data, either source of information could be utilized. Data collected by the two different instruments within different periods of time could be combined together and missing information in one source could be completed from the other. Nevertheless, in a data seems to be merely relying on self-report in both questionnaire and medical records such as socioeconomic status and socioeconomic problems, the questionnaire could provide the more trustable source of information. Also in some acute or minor illnesses that can't be directly linked to the reason of hospital admission the questionnaire is more trustable. Whereas, for sensitive (such as psychiatric history) or sophisticated medical information and in information that do not rely so much on self-report, medical records provide the most reliable source.

One of the limitations of the present study is that we have reviewed only admissions related to poisoning exposures. Other patients' records of previous admissions at the same hospital were not reviewed, as well as their other possible records elsewhere. Moreover, in our questionnaire we didn't ask about specific acute or chronic illness, but we asked generally (Did you see a doctor or psychiatrist recently during the last two months?); (Did you suffer any chronic illness?). Then the respondent was able to report only common chronic illness such as diabetes and hypertension as well as reporting their acute illness without emphases on identifying the right diagnosis.

## Conclusion

In conclusion, the present study has shown excellent to good agreements between the two sources of information on the majority of items related to marital status, health problems and history of previous poisoning and slight to poor agreements on most of the socioeconomic problems except for "boy-girl friend problems" (fair agreement).

## Competing interests

The author declares that he has no competing interests.

## Authors' contributions

AIF designed the study, analyzed the data, wrote the draft, reviewed the whole manuscript and approved it.

## Authors' information

The author, M.Sc. Clinical Pharmacy (Toxicology); formerly was the head of the Department of Pharmaceutical Services, the General Directorate of Pharmacy, Ministry of Health- Khartoum State, Sudan. Currently he is a Research Fellow of the Universiti Sains Malaysia (PhD candidate at School of Pharmaceutical Sciences) and attached to the National Poison Center of Malaysia.
